# Behavioral and neuropsychological profile of a male patient with mosaic PCDH19 mutation

**DOI:** 10.1016/j.ebr.2022.100559

**Published:** 2022-07-02

**Authors:** Margret Johannessen, Ida Marie Kjellsen, Eva Malt

**Affiliations:** a Department of Adult Habilitation, Akershus University Hospital, Akershus, Norway; b Institute of Clinical Medicine, Campus Ahus, University of Oslo, Oslo, Norway

**Keywords:** PCDH19, Mosaic male, Epilepsy, Executive functioning, Neuropsychological assessment

## Abstract

Mutations in the protocadherin 19 gene (*PCDH19)* are associated with a female-restricted form of epilepsy. As the disorder has an X-reversed inheritance pattern, men are usually healthy carriers. PCDH19 epilepsy among men due to mosaicism is rare but probably underdiagnosed. We describe the longitudinal development of the behavioral and neuropsychological profile of a male with a mosaic *PCDH19* mutation based on assessments carried out at 9, 11, 15, and 19 years of age. He was diagnosed with mild intellectual disability and autism spectrum disorder and had marked deficits in so-called *hot* executive functions related to emotion, as opposed to purely cognitive aspects of executive functions, the so-called *cold* executive functions. Although his epilepsy improved and cognition remained stable, executive dysfunction and behavioral problems became the prominent clinical features with age.

## Highlights

• Behavioral symptoms increased with age in a mosaic male with PCDH19 mutation.
• Neuropsychological assessment indicated deterioration of cognitive abilities.
• Executive dysfunction included impaired emotional control in social behavior.
• The orbitofrontal-subcortical network may be specifically affected.
• Deficit in executive dysfunction behaviors that require emotional awareness and regulation, empathy and Theory of mind may be associated with perceived low quality of life.

## Introduction

Mutations in the protocadherin 19 gene (*PCDH19)* predominantly cause epilepsy, intellectual disability, and behavioral disturbances. The clinical features resemble those of Dravet Syndrome [1]. Different names have been used to facilitate clinical identification of the disorder, including “Girls Clustering Epilepsy” (PCDH19- GCE) [2], “Early Infantile Epileptic Encephalopathy-9” (EIE19), and “PCDH19 Clustering Epilepsy” (PCDH19-CE) [3]. Here, we refer to the disorder as PCDH19-CE. The protein encoded by the gene is found in the central nervous system [4]. The location of *PCDH19* on chromosome Xq22.1 indicates X-linked female inheritance.

Together with the other non-clustered delta2 protocadherins, PCDH19 seems to be involved in the maintenance and plasticity of the adult hippocampal circuit [5]. Dysfunctional axon outgrowth and synaptic connections is hypothesized as the potential etiology of cognitive dysfunction in mutation carriers [6]. Cellular interference has been hypothesized to explain the inheritance pattern in which heterozygous females are affected and hemizygous males are healthy. However, over the last decade, men with mosaic *PCDH19* mutations have been found to possibly be affected. The first mosaic affected male for a pathogenic *PCDH19* variant was described in 2009 [7], and approximately 20 mosaic males had been described in the research literature as of 2020 [3].

The neuropsychiatric profile in females is highly heterogeneous, with intellectual disability ranging from mild to profound and psychiatric disturbances that feature executive dysfunction, hyperactive and attention deficit (ADHD), and obsessive–compulsive disorder (OCD) [8]. The overall neuropsychiatric profile in mosaic male patients is similar to female PCDH19-related phenotypes [3], [9], [10]. However, a full neuropsychiatric and neuropsychological profile has not yet been determined for a mosaic male. Due to the heterogeneity of the patient group, a full neuropsychological assessment is valuable for assessing each individual and provide customized treatment, as well as being necessary to learn about the constituent phenotype at a group level. Increased knowledge about the disorder will enable healthcare providers to intervene at an early stage of development to prevent and address developmental abnormalities and comorbidities and contribute to determining appropriate social and environmental interventions. The aim of this case report is to describe longitudinal clinical features, with a specific focus on the neuropsychological profile, in a male patient with mosaic mutation in the PCDH19 gene to contribute to understanding this rare genetic disorder.

## Methods

### Case description

We reviewed the clinical files of a patient with Caucasian European ethnicity who underwent neuropsychological assessment at the age of 19 years.

### Mutation analysis

Blood samples were obtained from the consenting patient and his parents. High-throughput sequencing and Sanger sequencing were performed at 18 years of age. The results indicated that the patient is mosaic for PCDH19 c.1068_1071delinsCA (p.Glu357Serfs*18), which is consistent with the patient’s phenotype. This variant had not yet been described. The variant destroys the open reading frame and introduces a premature stop codon in the mRNA of *PCDH19*. This probably leads to degradation of the mRNA in affected cells.

## Case description

### Early development and epilepsy

The patient was the second child of non-consanguineous parents. Pregnancy and delivery were uncomplicated. His familýs past medical history was unremarkable. The patient’s early psychomotor development was normal except for slightly delayed expressive (word and sentence) and receptive (understanding) language development. He could speak single words at the age of 2 years and sentences by the age of 3 years. From 3 to 4 years of age, he had episodes with hyperventilation, breath-holding, and facial flushing. There was concern about his social skills in kindergarten, as he withdrew from the other children and often exhibited anger in interactions. After starting school at the age of 6 years, he would often refuse to do the tasks required, showed clear signs of rigidity, and could react in a forceful manner if something did not turn out exactly the way he had foreseen. He could suddenly leave situations without taking his belongings with him.

At the age of 9 years, he had 1–2 seizures a week that were described as absences lasting about 5 s and episodes of impaired consciousness with involuntary urination. Repeated electroencephalograms (EEGs) under standard conditions showed normal findings without epileptiform activity, and magnetic resonance imaging (MRI) did not show any abnormalities.

At 10 years of age, he had several weekly seizures described as absences followed by a couple of seizure-free months. He also had seizures with staring, altered awareness, and automatism, such as rubbing of the hands and lip-smacking. Sometimes he reported olfactory hallucinations (unpleasant smell) as the first symptom of a seizure. During the seizures, he felt he was in a dreamy state with ripples in the stomach and felt as if he was crying. Sometimes he could hear what was happening around him. These seizures lasted for 5 to 30 s. Postictally, he often had a headache. The seizures were classified as focal aware seizures and focal impaired awareness seizures with non-motor onset. On one occasion, 1-hour EEG monitoring showed a short-lived interictal outbreak with paroxysmal theta-delta activity and sharp waves in the frontal and central parts of the left hemisphere compatible with possible focal epileptiform discharge. However, 48-hour video EEG during which the patient had impaired awareness showed no epileptic discharge. Carbamazepine was initiated at 17 mg/kg/day (blood concentration 30 µmol/L; reference 15–45 µmol/L) and later increased to 20 mg/kg/day. Seizure frequency increased, and the medication was changed to oxcarbazepine at 33 mg/kg/day (83 µmol/L; reference 45–140 µmol/L). The seizure frequency did not change and the epilepsy diagnosis was considered uncertain. Therefore, the oxcarbazepine was discontinued.

At 16 years of age, the patient’s seizure frequency increased after a period with low frequency. He presented with focal impaired awareness seizures with non-motor onset, and sometimes motor onset, interpreted as temporal lobe epilepsy. He also had generalized tonic-clonic seizures, some of them focal to bilateral tonic-clonic seizures, with 1 to 2-minute duration. The seizures were often triggered by mental stress. Afterwards, he was sleepy and confused for 10 min, but full restoration could take several days. He usually had several seizures within 1 or 2 days, followed by a seizure-free month. EEG under standard conditions showed multifocal epileptiform discharges with left hemispheric predominance. He received up to 1200 mg/day valproate (568 µmol/L; reference 300–600 µmol/L), which reduced the frequency of focal seizures but not the generalized tonic-clonic seizures. Adding 50 mg/day lamotrigine (11.6 µmol/L; reference 10–60 µmol/L) had no effect on seizure frequency and was discontinued after 2 years. Valproate was gradually reduced to 450 mg/day (173 µmol/L) due to somnolens and depression. He became more alert without any increase in seizure frequency.

At 19 years of age, 3-day video-EEG during symptoms of stomach upset showed no definitive EEG correlations. EEG showed abundant sporadic epileptiform discharges in both frontotemporal regions, with right-sided predominance. Clobazam was added at 10 mg/day (0.78 µmol/L; reference 0.1–1 µmol/L), and the interval between seizures increased. Valproate was increased to 600 mg/day (238 µmol/L) and was well tolerated.

Currently, 25 years old, the seizures still come in clusters within 1 to 2 days, followed by seizure-free periods lasting up to 8 months. One or two days before a bilateral tonic-clonic seizure, the patient is nervous and complains of stomach upset and forgetfulness. The following days he has several hypomotor seizures with staring and behavioral arrest lasting 15–20 s. He is regularly afflicted by anxiety, aggression, and affect lability and has great difficulties participating in work-related or other organized activities.

### Neuropsychological assessment

The proband underwent formal psychological assessments four times, at the ages of 9, 11, 15, and 19 years (Table 1). The first clinical evaluation was conducted at the Children’s Clinic when he was a 9-year-old due to suspected neurological disease. The same year, the Educational and Psychological Counselling Service briefly assessed him due to suspected difficulties in concentration and difficulties in participating in social situations at school. The clinical evaluations when he was 11 and 15 years old were conducted at the Department of Child Habilitation, where he was followed up until the age of 18. The last evaluation at 19 years of age (Table 2) was conducted at the Department of Adult Habilitation, where he still receives follow-up.

**Table 1:** Neuropsychological results across assessments at 9, 11, 15, and 19 years old.

Domain of function	9 years	11 years	15 years	19 years

**Effort**				
**Intellectual functioning**				
Verbal	−1.5 SD WISC-III	− 3 SD WISC- III	− 3 SD WISC-IV	− 2 SD WAIS- IV
Visual	− 0.5 SD	− 2 SD	− 1.5 /− 2 SD	− 1.5/ − 2 SD
Leiter-R		− 2 SD		
Stanford-Binet		− 2 SD		
**Attention and working memory**				
Digit span	− 1 SD	**	**	*
Spatial span		Average		
Connors CPT-III		Average		*
CAVLT/CVLT-II 1. trial		**	**	Average
Arithmetic	Low average	**	**	Low average
**Visual spatial skills**				
VMI		Impaired		Impaired
Rey Complex Figure		Impaired	− 2 SD	*
**Memory**				
Total immediate recall		Impaired CAVLT-II	Impaired	Impaired CVLT- II
- Short delay		Impaired	Impaired	*
- Long delay		Impaired	Impaired	*
WNS logical memory				
- Immediate recall		Average		
- Delayed recall		Average		
Rey Complex Figure				
- Short delay		Impaired	−2 SD	*
- Long delay		Impaired	Impaired	*
NEPSY memory for faces		Average	Impaired	
Memory for designs		Impaired		
**Processing speed**				
Coding	Impaired	**		Low average
D-KEFS trails numbers		Low average	Average	Low average
**Executive function**				
D-KEFS trails switching		Low average	Average	Low average
WCST			Average	Average
D-KEFS verbal fluency FAS		**		Average
NEPSY Tower of London		Impaired		
D-KEFS color-word interference test		**		Impaired
**Motor-sensory skills**				
Grooved pegboard test				
Right (dominant)		Average	Average	Low average
Left		Average		Low average
D-KEFS trails motor speed				Average
**Language**				
Boston Naming Test		−3 SD	−3 SD	
Token test		−3 SD	Low average	
Comprehension	Impaired	**	**	Low average
D-KEFS CW1 word reading		**		Impaired
NEPSY phonological processing		Low average		

SD: standard deviation.

* Indicates scoring was interrupted by the patient;

** results not available.

**Table 2:** Comprehensive neuropsychological assessment results organized by domain of function.

Domain of function	Score^a^	Interpretive range/effort
**Intellectual functioning**		
Wechsler Adult Intelligence Scale – IV (WAIS-IV)		
Verbal comprehension index	69	Impaired
Similarities	**4**	Impaired
Vocabulary	**6**	Low average
Information	**4**	Impaired
Comprehension	**6**	Low average
Perceptual reasoning index		
Block design	*	
Matrix reasoning	**4**	Impaired
Visual puzzles	*	
Working memory index		
Digit span	*	
Arithmetic	**7**	Low average
Letter number sequencing	*	
Processing speed index	82	Low average
Symbol search	**7**	Low average
Coding	**6**	Low average

**Attention and working memory**		
WAIS-IV		
Arithmetic	**7**	Low average
Digit span		
Forward	**4**	Impaired
Backward	**7**	Low average
Sequencing	*	
Delis- Kaplan Executive Function System (D-KEFS)		
Trail-making test		
Number-letter switching	**8**	Low average
California Verbal Learning Test (CVLT-II) 1 trial	T = 55	Average
Connors CPT-III	*	

**Processing speed**		
WAIS-IV		
Coding	**6**	Low average
Symbol search	**7**	Low average
Delis- Kaplan Executive Function System (D-KEFS)		
Trail-making test		
Visual scanning	**1**	Impaired
Number sequencing	**8**	Low average
Letter sequencing	**4**	Impaired
Number-letter switching	**8**	Low average
Motor speed	**11**	Average
Color-word interference test		
Color naming	**4**	Impaired
Word reading	**4**	Impaired

**Language/knowledge**		
Delis- Kaplan Executive Function System (D-KEFS)		
Verbal fluency test		
Letter fluency	10	Average
Category fluency	5	Impaired
Category switching	9	Average
WAIS-IV		
Information	4	Impaired
Vocabulary	6	Low average
Similarities	4	Impaired
Comprehension	6	Low average

**Memory**		
California Verbal Learning Test (CVLT-II)		
Total immediate recall	T = 35	Impaired
Short delay	*	
Long delay	*	
Recognition hits	*	
Rey Complex Figure		
Immediate recall	*	
Delayed recall	*	
**Executive function**		
Wisconsin Card Sorting Test	6/6 categories	Average
	T = 48	Average
D-KEFS		
Color-word interference test		
Inhibition	3	Impaired
Trail-making test		
Number-letter switching	8	Low average
Verbal fluency test		
Letter fluency	10	Average
Category fluency	5	Impaired
Category switching	9	Average
WAIS-IV		
Similarities	**4**	Impaired
Matrix reasoning	4	Impaired
**Visual spatial skills**		
Rey Complex Figure	*	
Copy	*	
VMI	T = 30	Impaired
WAIS-IV		
Block design	*	
**Motor sensory skills**		
Grooved pegboard test,		
Right (dominant)	T = 42	Low average
Left	T = 40	Low average

*indicates that the scoring was interrupted by the patient.

a Standard scores are in regular text, scaled scores are in bold;

At 9years old, the Wechsler Intelligence Scale for Children (WISC- IV) [11] revealed a full-scale intelligence quotient that was borderline for intellectual disability (Table 1). There was a discrepancy between his verbal and performance scores, with significantly lower verbal task scores than performance scores. During cognitive testing, he exhibited unease and, gradually, signs of hyperventilation with increased difficulty performing tasks. The results of the first assessment showed concentration difficulties and borderline cognitive function. At 11 years old, he presented with increased aggression and his social functioning with peers was problematic. A new assessment with the Stanford-Binet intelligence scales [12] had results 2 standard deviations below the average range. He was still assessed as performing weaker on the verbal tasks than the performance tasks. The Autism Diagnostic Interview (ADI-R) [13] showed qualitative impairment in the areas of social interaction and communication and the presence of repetitive and stereotypic behavior. The Vineland adaptive scale (VABS-II) [14] results were below what was expected for his age, particularly in the areas of social and communication functioning. The WISC-IV assessment was lower than 2 years prior, more than 2 standard deviations below the average range. Overall, the patient’s neuropsychological profile was divergent, with clear difficulties in language and visual perception. The Behavior Rating Inventory of Executive function (BRIEF) [15] showed that he struggled with working memory, initiating, planning, and monitoring. His parents described him as being dependent on routines, with poor flexibility and conversation skills. His behavior problems were assessed to be independent of his epilepsy. The results of the second assessment showed autism and mild intellectual disability (ICD-10 F84.0 and F70.0). At 15 years old, he was re-tested to monitor his developmental trajectory. The WISC-IV revealed an increased difference between verbal and visual abilities, and the results on attention tests fluctuated. His behavior problems during testing were striking for his age. The BRIEF indicated greater difficulties with executive functioning than on previous tests and revealed particular difficulties related to language and visual perception. His previous diagnosis of autism spectrum disorder (F84.0) and mild intellectual disability (F70.0) were maintained.

At 19 years old, a neuropsychological assessment was performed to investigate whether his development had deteriorated (Table 2). His behavior during testing was similar to previous assessments; he appeared to have little motivation and exhibited impulsive behavior with a lack of self-control, such as throwing some of the testing material at the assessor. He would often talk about other things and needed encouragement to stay focused on the tasks he was given. The WAIS-IV [16] results were in the range of mild intellectual disability. As seen earlier, the results of psychomotor and attention tasks fluctuated, and some tasks were not possible due to lack of cooperation. Results for tasks on executive functioning showed great variation, and his behavior during testing indicated specific difficulties in regulating behavior. His apparent lack of motivation during testing could raise questions about the validity of the test results. However, considering his lack of ability to endure when asked to preform mental tasks, the results are considered to substantiate difficulties with sustained attention. The VABS-II and BRIEF showed great difficulties related to flexibility, initiating, control of impulses and emotions, and planning. There were specific problems in executive functions, such as abstract reasoning, sustained attention, mental flexibility, and behavior inhibition. His repertoire of interests was slightly restricted, and he had substantial problems with social interaction, meeting the criteria for pervasive developmental disorder. At 24 years old, the Personal Wellbeing Index – Intellectual Disability (PWI-ID) [17] showed that he perceived his quality of life as very low in the areas of social life and work, spare time activity, and future prospects (4/10). He is in need of high-level assistance in his daily life, from caregivers and health care staff, to organize, follow through, and participate in work and physical activities.

## Discussion

This case is unique in that it illustrates the longitudinal development of epilepsy and the cognitive and neuropsychiatric profile in a male with a de novo mosaic variant of PCDH19. His first seizures were observed at 3–4 years of age, which is a little later than commonly described [6], [9], [18]. Repeated EEG examinations have shown epileptiform discharges in both frontotemporal regions, but no ictal activity has been identified. Focal seizures starting from the frontotemporal region are the most frequent seizure type in PCDH19-CE [6], [18]. The patient had both focal and generalized seizures. However, focal onset is often suspected in patients with bilateral motor seizures. As in other patients with PCDH19-CE a high proportion of his seizures occurs in clusters [6], [9], [18]. He also has had less frequent seizures but more behavioral disturbances with age. As in other patients with PCDH19-CE, carbamazepine has had no effect, and clobazam and valproate are the antiseizure medications that seem to have the best effects [6]. Unlike many patients with PCDH19-CE, our patient has no temperature sensitivity, and his seizures are not triggered by fever, but emotional stress [9], [18], [19]. His report of feeling as if he was crying may be interpreted as an affective symptom and varies from the fearful screaming often described [9], [18]. However, we cannot exclude that some of the events may have been psychogenic nonepileptic seizures, such as his “hyperventilation seizures”.

The patient’s general cognitive abilities seemed to deteriorate from 9 to 11 years of age because there was a decline in raw scores (Table 1). From 15 to 19 years of age, his general cognitive abilities seem to have remained stable, but this finding is more uncertain because different test batteries for intellectual functioning were used. The neuropsychological assessments from 9 to 19 years consistently showed linguistic and communication difficulties, difficulties with visual perception, fluctuating attention, and dysexecutive functioning. An increasing gap between verbal and visual abilities was found until 15 years old. At 19 years old, the difference between verbal and visual abilities was less apparent. However, the patient was less cooperative and more difficult to examine. Therefore, it was difficult to conclude whether this finding was related to a worsening of his executive functioning or because his verbal and visual abilities had converged. Due to the behavior problems, the results must also be considered more uncertain. During his childhood, the conclusion was that the behavior problems were independent of the epilepsy. His increasing anxiety, aggression, and affective symptoms were later considered to likely be related to the epileptic picture.

Few articles have described the full cognitive profiles of both female and male patients with mosaic *PCDH19* mutations. The degree of mental retardation is mostly found to range from normal/borderline to moderate intellectual disability [9]. In addition to obsessive and hyperactive traits, frontal lobe dysfunction has been suggested in the cognitive and behavioral profiles of females with epilepsy and *PCDH19* mutation [19]. Males with mosaic variants of PCDH19-CE have been described as having behavioral and psychiatric disturbances, such as autism, aggression, behavioral problems, ADHD, rigidity, irritability, anxiety, mood disturbances, short attention span, and various degrees of delay in language development [9], [10].

The findings from this case description indicate specific executive function deficits (deficit in organizing, planning, impulse control, and abstract reasoning) in addition to intellectual disability and pervasive developmental disorder. Dysexecutive syndrome has also been found to be related to *PCDH19* mutation [19]. The prefrontal cortex is traditionally linked to executive functions [20]. In the literature, a distinction is commonly made between *cold* and *hot* executive functions. *Cold* executive functions are associated with the dorsolateral prefrontal cortical regions and include planning, cognitive flexibility, working memory, behavioral monitoring, and inhibition. *Hot* executive functions are mediated by the ventromedial and orbito-frontal cortices, which support behaviors that require emotional awareness and regulation, empathy, and theory of mind [20]. The anterior and posterior cingulate cortices are also involved, and differences in prefrontal cingular networks may explain different neuronal correlates of *hot* versus *cold* executive functions [21].

The patient had EEG findings involving the frontal-temporal areas of the left hemisphere, which is in accordance with previous studies of the electro-clinical pattern in PCDH19-CE patients presenting with epileptogenic dysfunction related to frontotemporal structures and the limbic system [18], [22]. We suggest that this epileptogenic dysfunction represents a physiological correlate to the functional deficits in *hot* executive functions. Interestingly, the executive tasks that were presented in structured circumstances to assess the *cold* components of executive function showed higher levels of functioning.

Executive dysfunctions are among the most prevalent neurodevelopmental features associated with autism spectrum disorder (ASD) [23]. The current evidence is inconclusive, but groups with ASD seem to perform significantly worse than matched controls on all measures of *cold* and *hot* executive functions [24]. Although our patient’s trajectory of neuropsychological development may be attributed to autism spectrum disorder, as well as his epilepsy syndrome, it is tempting to suggest that dysfunction of the frontal control system-limbic system in association with disturbances in *hot* executive functions may be a hallmark of PCDH19-CE. This has important implications, as deficits in emotional executive function are associated with reduced quality of life [25]. Therefore, we suggest that the proband’s perception of very low quality of life is associated with the deficits in *hot* executive functions.

## Conclusion

This case study documents the development of epilepsy and neuropsychological functions in a male with a mosaic *PCDH19* mutation from a longitudinal perspective. He shows marked deficits in *hot* executive functions that support behaviors requiring emotional awareness and regulation, empathy, and theory of mind. Our findings illustrate that a broad neuropsychological assessment, including standardized measures and questionnaires, is needed to detect specific defects in executive functioning with important consequences for daily functioning, quality of life, and clinical care. Environmental adjustments and targeted interventions may be needed. Further studies are needed to clarify whether dysfunction of the frontal control system-limbic system in association with disturbances in *hot* executive functions may be a hallmark of PCDH19-CE in males.

## Ethical approval

We confirm that this report is consistent with the ethical publication guidelines.

## Compliance with ethical standards

*Ethical approval:* All procedures performed in the study involving human participants were in accordance with the ethical standards of the institutional research committee and with the 1964 Helsinki declaration and its later amendments or comparable ethical standards.

*Informed consent:* Informed consent was obtained from the e participant and his next-of-kin.

## Funding

This study was funded by Akershus University Hospital.

## Declaration of Competing Interest

The authors declare that they have no known competing financial interests or personal relationships that could have appeared to influence the work reported in this paper.
